# Does high-speed rail stimulate university technology transfer? evidence from China

**DOI:** 10.1371/journal.pone.0285431

**Published:** 2023-05-19

**Authors:** Xiao Wu, Haohan Luo, Ying Wu

**Affiliations:** 1 Party Committee Office, Southwestern University of Finance and Economics, Wenjiang, Chengdu, Sichuan Province, China; 2 School of Finance, Southwestern University of Finance and Economics, Wenjiang, Chengdu, Sichuan Province, China; 3 School of Business, Nanjing Normal University, Nanjing, Jiangsu Province, China; Shenzhen University, CHINA

## Abstract

Prior studies ignore the impact of infrastructure on university technology transfer. High-speed rail, China’s most significant infrastructure, has played an essential role in the economy and society. Using high-speed railway construction as a quasi-experiment and a large sample of Chinese universities for the 2007–2017 period, we investigate the impact of high-speed rail on university technology transfer. We provide extensive evidence that high-speed rail has a positive effect on university technology transfer. The finding remains valid after a battery of robustness tests. Mechanism tests find that high-speed rail can improve university technology transfer by promoting the interaction between universities and enterprises and improving enterprises’ technology demand for universities. Further analysis shows that better intellectual property protection strengthens the effect of high-speed rail on university technology transfer, and the relationship between high-speed rail and university technology transfer is more prominent in the regions with underdevelopment technology trading markets. Our study suggests that high-speed rail is an important variable that affects university technology transfer.

## 1. Introduction

Technologies advanced is a vital drive forcing economic and environmental improvements [1]. University is the primary producer of technological advanced. University technology transfer helps technologies achieve economic value, which affects regional economic development [2–4] and has been grabbing the substantial attention of both policymakers and scholars [5–8]. Meanwhile, technology transfer also helps universities to obtain incomes and strengthens their general reputations to attract the brightest students and researchers [6,9]. More important, in the background that the COVID-19 pandemic hit the global economy [10–13], the successful transformation of university technology can help to reduce the adverse effect of the pandemic [14].

How to stimulate university technology transfer has become a hot topic. Prior studies, however, pay more attention to the impact of formal and informal mechanisms on university technology transfer, such as university science parks [15], university-industry cooperation research centers [16], university technology transfer offices [17], university incubators [18], government regulation [19], inventors’ technology service [20], and culture [21]. This no doubt that infrastructure construction impacts the development of the economy and society [22–24]. However, we know little about the impact of infrastructure on university technology transfer. To fill the gap, we investigate whether high-speed rail(HSR) affects university technology transfer.

China began the construction of HSR in 2008 and had been paying considerable investments in the HSR to develop an HSR system across the country. The total operating mileage of HSR exceeded 39 thousand kilometers in 2020, more than two-thirds of the world’s total HSR mileage, and covers over 80% of prefecture-level cities. HSR can operate at the maximum speed of 300 km/h, which produces a space-time compression effect, significantly increases transport accessibility for the cities along with the HSR network, and facilitates the mobility of production factors. HSR has been affecting both economically and socially [22–30]. To our limited knowledge, the paper examines the impact of the HSR on university technology transfer for the first time.

HSR could affect university technology transfer through a few plausible channels. First, university technology includes explicit knowledge and tacit knowledge. Tacit knowledge plays a vital role in technology transfer [31] and is difficult to represent on paper, store electronically, or transfer to others [32]. This makes the inventors’ face-to-face interactions with consumers significant in university technology transfer [20]. HSR reduces geographic proximity, shortens travel time, and accelerates information sharing and face-to-face interactions [22]. For example, Dong et al [33] find that HSR promotes face-to-face interactions between skilled workers and knowledge diffusion and idea spillovers. Therefore, the HSR can facilitate the mobility of tacit knowledge between inventors in universities and skilled workers in enterprises, which is conducive to university technology transfer. We term this channel as *the interaction channel*.

Second, HSR can affect university technology transfer by expanding the demand for university technology. HSR makes a substitution impact on passenger traffic on conventional trains, providing more lines for freight transportation and improving market integration [34]. This helps reduce enterprises’ operating costs and increases the attractiveness for enterprises to operate in regions with HSR. For example, prior studies have pointed out that HSR promotes industry agglomeration [35,36]. The agglomeration of the industry means that enterprises’ competition will improve. To obtain a competitive advantage, enterprises need advanced technologies to improve the production process [37]. Besides, technologies developed by universities rely on a careful discussion of earlier research results, including careful documentation of error and trial, which are hard for firms to generate internally [37]. Enterprises can directly exploit technology outputs developed by universities, which reduces research and development time and is conducive to obtaining a competitive advantage. Therefore, HSR can increase industry agglomeration, improving the competition in the region, which expands the demand for the technologies of universities. We term the channel as the *demand* channel.

Based on the above analysis, we expect that the HSR will have a positive effect on university technology transfer. To conduct our study, we collect detailed information on the Chinese HSR line from the Chinese Research Data Services (CNRDS). The data regarding university technology comes from the Chinese Ministry of Education. Due to HSR construction as a quasi-experiment, using the staggered Difference-in-Difference (DID) method and a total of 6114 university-year observations representing 732 universities from 2007 to 2017, we document extensive evidence that HSR promotes university technology transfer. The finding is robust to the alternative regression method, excluding universities without technology transfer in the sample period, replacing the measure of technology transfer, controlling the effect of government regulation, and testing the parallel trend hypothesis of DID method. In terms of mechanism tests, we find that HSR promotes university technology transfer by improving the interaction between universities and enterprises and expanding the demand for university technology. Heterogeneity analysis shows that better intellectual property protection strengthens the effect of HSR on university technology transfer, and the relationship between HSR and university technology transfer is more prominent in the regions with underdevelopment technology trading markets.

Our paper contributes to the literature in three ways. First, we enrich the literature on factors affecting university technology transfer [17,38,39]. This paper finds that infrastructure, namely, HSR is an essential factor affecting university technology transfer. Second, this paper expands the understanding of the effect of HSR [23,26,28,29]. The evidence in this paper suggests that HSR has a positive effect on university technology transfer. Finally, this paper expands the research on university technology transfer in developing countries [40–42].

The rest of the paper is organized as follows. Section 2 provides a literature review and the background of Chinese university technology transfer. Section 3 represents the sample, variables, and empirical designs. Section 4 reports the empirical results. Section 5 concludes.

## 2. Literature review and university technology transfer in China

### 2.1 Literature review

Our paper builds on two strands of literature: recent studies that focus on what factors affect university technology transfer and research on the effect of high-speed railway.

Prior studies argued that formal factors could impact university technology transfer, such as strategic choice [38], university-industry cooperation research centers [16], university technology transfer offices [17], university incubators [18], government regulation [19], and research outcome quality [39]. Besides, the informal factors also play an important role in university technology transfer, including developed factor market [43], inventors’ technology service [20], geographic reach [44,45] and culture [21]. However, no existing paper examines whether infrastructure construction affects university technology transfer.

HSR is an important infrastructure construction in recent years and has a great effect on the economy and society. Scholars have found that HSR has a positive effect on regional economic growth [25,26], asset prices [23,46], industry agglomeration [35], air quality [29], job opportunities [24], entrepreneurship [22, consumption density [47], and tourism development [30], corporate innovation [28], and analyst forecasts accuracy [48]. However, HSR also aggravates population loss in shrinking cities [49] and increases the cost of debt [27]. Therefore, the effect of HSR is a double-edged sword.

Based on the above literature review, we can find that although HSR has been reshaping the economy and society in China, the extant paper ignores the role of HSR in university technology transfer. This paper helps fill the gap and investigates the effect of HSR on university technology transfer.

### 2.2 University technology transfer in China

After the establishment of The People’s Republic of China in 1949, China built a modern university system. University technology transfer can be divided into two stages. From the 1950s to the early 1980s, China was a centrally planned economic system, and the government determined universities’ technology development and transfer. Universities obtained research funds from the government, and research activities serve the government development goal, such as agricultural and national defense. Technology and knowledge were not merchandised in the marketplace, and the government owned all intellectual property [5]. Besides, all industries also were controlled by the government. Technology transfer from universities to the industry was in a system government-dominated.

University technology transfer has come into the second stage since 1979. China conducted the most important reform in 1979, namely, transforming its national economic system from a planned to a market-orientated economic system. China began to strengthen economic development and argued that scientific research should serve the development of the national economy. To stimulate technology application in economic development, China conducted a series of reforms in university technology transfer. In 1986, the government changed the fund system and opened the technology market. Universities were allowed to undertake research projects from other sources and obtain profit. In 1987, China issued the Technology Contract Law to guarantee technology contract parties’ lawful rights, which promotes the growth of the technology market. One milestone in university technology transfer happened in 1996; China enacted the Scientific and Technological Progress Law and encouraged universities to expand cooperation with enterprises, including private firms. More importantly, the law asked universities to provide an incentive for inventors, who can obtain no less than 20% of technology transfer income. The law is regarded as the Chinese “Bayh-Dole Act” [7]. In 1999, the Chinese Ministry of Education gave universities intellectual property of technology outputs to promote university technology transfer. In 2001, the Ministry of Education and the State Economic and Trade Commission jointly established state technology transfer centers in six universities. In 2007, the Chinese State Taxation Administration removed tax for university technology transfer. To further stimulate technology transfer, in 2015, China amended the Scientific and Technological Progress Law that provided more incentives for university technology transfer.

China has established integrated university technology transfer systems, such as science parks, technology transfer offices, and university-industry cooperation centers. It is worth noting that China developed an additional form, namely, university-affiliated enterprises [50]. Although China makes efforts to improve university technology transfer, technology transfer in universities is lower. According to the data from the Chinese Ministry of Education in 2017, the number of patents granted was 144375 in universities, but the number of patents sold to enterprises was 4803, which accounts for only approximately 3.3% of the total patents. How to improve university technology transfer is vital for all stakeholders.

## 3. Sample and empirical design

### 3.1 Sample

To construct our sample, we start all Chinese Universities from 2007–2017. The data of universities is collected from the University Science and Technology Statistics issued by the Chinese Ministry of Education. The report includes six major categories of universities’ research activities, including employees, science and technology funds, science and technology projects, technology output and transfer, and awards. In addition, each category is further divided into subcategories, and the corresponding statistical data are given. Besides, considering some universities that changed their names, we merge these samples. The data on HSR is collected from the Chinese Research Data Services (CNRDS) database, which provides the line name and opening date of HSR. Other data is collected from the CNRDS database and the China Nation Intellectual Property Administration. Then, we exclude universities-year observations with missing information for variables used in the paper. Our final sample includes 6114 university-year observations representing 732 universities. Finally, to mitigate the effect of outliers, we also winsorize all continuous variables at the 1% and 99% levels.

### 3.2 The measure of high-speed railway

The opening time of HSR in different prefecture cities is heterogeneous. Following Zhang et al [28], we use a dummy variable to indicate whether a prefecture-level city where universities are located opens high-speed rail. Specifically, if the prefecture-level city where the university i is located has constructed the high-speed rail in year t, the HSR has a value of 1 in the year t and after and 0 otherwise.

### 3.3 The measure of university technology transfer

The number of patents, transfer contracts, and transfer revenues are used to measure university technology [43,51]. However, the number of patents is one of the measurements of technology outputs and fails to reflect the technology transfer from university to industry. Therefore, following Wu et al [52], we use the number of transfer contracts to measure university technology transfer. Duo to the right skewness of the number of license agreements, we use the natural logarithm of one plus transfer contract counts to measure university technology transfer (Transfer). In robustness tests, we use the natural logarithm of one plus transfer incomes to measure university technology transfer (Transfer1).

### 3.4 Empirical model

HSR construction is regarded as a quasi-experiment. To examine whether HSR affects university technology transfer, following Wang et al [27], Zhang et al [28], and Wu et al [53], we use the staggered Difference-in-Difference method. The method is used to examine the effect of policy shocks happening at different times.


$$\begin{array}{cc} {Transfer}_{i,t} & =\beta_{0}+\beta_{1}{HSR}_{i,t}+\beta_{2}{Treat}_{i}+\beta_{3}{Resp}_{i,t}\\ & +\beta_{4}{Type}_{i,t}+\beta_{5}{Expenditure}_{i,t}{+\beta_{6}AGDP}_{i,t}\\ & +\beta_{7}{Digital}_{i,t}+\eta_{j}+\mu_{t}+\varepsilon_{i,t} \end{array}$$ (1)

Where Trnsfer _i,t_ is university technology transfer of the university i in year t. HSR is a dummy variable, indicating whether a prefecture-level city where the university i is located has HSR in year t. Treat is a dummy variable that equals 1 when a prefecture-level city has a high-speed railway in the sample period and 0 otherwise. We also control other factors that may affect university technology transfer. Specifically, O’Shea et al [54] argued that human capital plays a vital role in university technology transfer; thus, we use the natural logarithm of the number of university researchers to control the effect of human capital (Resp) [52]. Universities can be divided into two types, namely, leading universities and generally universities. Leading universities obtain more financial support from the government and have a higher reputation in society, which might affect technology transfer [43]. We use a dummy variable to indicate whether a university belongs to a leading university (Type). Specifically, if a university is in the “211” project, the university is defined as a leading university, suggesting that Type is 1 and 0 otherwise [52]. Besides, we also consider the economic environment where a university is located.

Government expenditure is one of the significant factors affecting the economy [55]. We use the natural logarithm of government expenditure in the province where a university is located to control the effect of government (Expenditure). For example, the government may provide subsidies for university technology transfer. We use the natural logarithm per capital of gross domestic product (AGDP) in the province where a university is located to control the effect of economic development. Considering the fact that digital economy is reshaping the Chinese economy and society [56–59], we control the effect of the digital economy. For example, the digital economy may provide convenience for the interaction between universities and enterprises, which may promote technology transfer. Specifically, we use the natural logarithm of the users of the internet to measure digital economy (Digital) because the internet is the base of the digital economy. Finally, we also include province-fixed effects to control unobservable time-invariant region-specific characteristics(η) and year-fixed effects to control common time trends (μ). The variables are defined in Table 1.

**Table 1:** Variable definitions.

Variable	Definition
HSR	If the prefecture-level city where the university i is located has constructed the high-speed rail in year t, the HSR has a value of 1 in the year t and after and 0 otherwise
Transfer	The natural logarithm of one plus transfer contract counts [52]
Treat	A dummy variable that equals 1 when a prefecture-level city has a high-speed railway in the sample period and 0 otherwise.
Resp	The natural logarithm of the number of university researchers [52]
Type	A dummy variable that equals 1 if a university is in the “211” project and 0 otherwise [52]
Expenditure	The natural logarithm of government expenditure in the province where a university is located
AGDP	The natural logarithm per capital of gross domestic product in the province where a university is located
Digital	The natural logarithm of the user of the internet in the province where a university is located

## 4. The empirical results

### 4.1 Descriptive statistics

Table 2 reports the summary statistics on the variable used in our analysis. The mean value and maximum of Transfer are 1.0949 and 5.5053, respectively, suggesting that the rate of technology transfer has a more significant difference among universities. Besides, the mean value of HSR is 0.5592, indicating that observations after the opening of HSR account for 55.92%. Finally, the minimum value and maximum value of Digital are 5.53891 and 8.8937, showing that the development of the digital economy is uneven across the country, which is consistent with previous studies [57–59].

**Table 2:** Descriptive statistics.

Variable	N	Mean	Std	Minimum	Median	Maximum
Transfer	6114	1.0949	1.4734	0.0000	0.0000	5.5053
HSR	6114	0.5592	0.4965	0.0000	1.0000	1.0000
Treat	6114	0.8873	0.3162	0.0000	1.0000	1.0000
Resp	6114	5.4235	1.3902	1.0986	5.5530	8.4998
Type	6114	0.1649	0.3711	0.0000	0.0000	1.0000
AGDP	6114	10.5585	0.5198	9.3959	10.5690	11.6531
Digital	6114	7.4416	0.6994	5.3891	7.4714	8.8937
Expenditure	6114	8.0980	0.6158	5.4883	8.1547	9.5064

The table reports the descriptive statistics for university technology, HSR, and control variables. The sample period is from 2007 to 2017. The sample size is 6114.

### 4.2 The Person correlation matrix

Table 3 reports the individual correlation coefficients between variables. We can find that the correlation coefficient between HSR and Transfer is positive and significant at the 1% statistical level. In addition, the correlations between HSR and the control variables are not high, suggesting that multicollinearity is not a severe problem.

**Table 3:** The table represents the correlation coefficient between variables.

	Transfer	HSR	Resp	Type	Expenditure	AGDP	Digital
Transfer	1						
HSR	0.194***	1					
Resp	0.494***	0.154***	1				
Type	0.400***	0.124***	0.448***	1			
Expenditure	0.115***	0.477***	0.059***	-0.024***	1		
AGDP	0.159***	0.531***	0.177***	0.140***	0.630***	1	
Digital	0.120***	0.401***	0.049***	-0.075***	0.905***	0.512***	1

The sample period is from 2007 to 2017. The sample size is 6114. The symbols *, **, and *** denote significant levels at the 10%,5%, and 1%, respectively.

### 4.3 Main results

In this subsection, we use model (1) to investigate the impact of HSR on university technology transfer. As shown in column 1, we only regress Transfer on HSR. The coefficient of HSR is positive and significant at the 1% statistical level, suggesting that HSR has a positive effect on university technology transfer. In column 2, we control the effect of university characteristics, such as human capital and universities’ type. The coefficient of HSR is significantly positive. In the last column, we put all control variables, and the coefficient of HSR is positive and significant at the 1% statistical level, indicating that HSR promotes university technology transfer.

In terms of the control variables, we find that the coefficient of Resp is positive and significant at the 1% statistical level, suggesting that more substantial universities’ human capital promotes university technology transfer, which is consistent with the finding of O’Shea et al [54]. Besides, we find that leading universities have higher technology transfer [43] because leading universities usually have a better reputation in education and academics. Finally, we fail to find that the development of digital economy affects university technology transfer.

### 4.4 Robustness tests

In this subsection, we run several robustness tests. First, according to descriptive statistics, we can find that the median value of Transfer is 0, indicating that many university-year observations have zero technology transfer. To address the issues that university technology transfer is nonnegative and discrete, following Fisch et al [60], we replace OLS regression with negative binomial regression in column 1 of Table 4. The coefficient of HSR is positive and significant at the 10% statistical level, supporting the finding that HSR promotes university technology transfer.

**Table 4:** The table reports the impact of HSR on university technology transfer.

	(1)	(2)	(3)
	Transfer	Transfer	Transfer
HSR	0.334***	0.173***	0.175***
	(6.247)	(3.702)	(3.733)
Treat	0.508***	0.208***	0.206***
	(10.507)	(4.465)	(4.418)
Resp		0.405***	0.405***
		(33.149)	(33.182)
Type		0.869***	0.868***
		(15.640)	(15.637)
Expenditure			0.066
			(0.244)
AGDP			-0.1930
			(-0.680)
Digital			-0.2410
			(-1.491)
Constant	1.273***	-1.359***	1.767
	(9.655)	(-10.714)	(0.753)
Province fixed effect	Yes	Yes	Yes
Year fixed effect	Yes	Yes	Yes
N	6114	6114	6114
R^2^	0.124	0.352	0.352

The sample period is from 2007 to 2017. The sample size is 6114. The t-statistics are reported in parentheses. The symbols *, **, and *** denote significant levels at the 10%,5%, and 1%, respectively.

In the Chinese university system, some universities are positioned to train applied talents and pay more attention to teaching than research. These universities fail to transfer any technology in the sample period, which may cause bias in our study. In column 2 of Table 4, we exclude the universities that have zero technology transfer in our sample period. We find that the coefficient of HSR is significantly positive, which is consistent with the finding in Table 3.

Following Siegel et al [51] and Baglieri et al [17], we use technology transfer incomes to measure university technology transfer. Specifically, we use the natural logarithm of one plus technology transfer incomes (Transfer1) [52]. As shown in column 3 of Table 4, the coefficient of HSR is positive and significant 1% statistical level, suggesting that HSR has a positive effect on university technology transfer.

In column 4 of Table 4, we consider the impact of government regulations on university technology transfer. In 2015, China amended the Scientific and Technological Progress Law, which expanded the universities’ power of research results obtained with government funding and increased the reward standard from 20% to 50% for inventors in technology transfer. To control the impact of the law on university technology transfer, we put a dummy variable in our regression (Law). Law is 1 after 2014 and 0 otherwise. We can find that HSR has a positive effect on university technology transfer after considering the impact of the Law. Besides, the coefficient of Law is insignificant, suggesting that the law may fail to stimulate university technology transfer in China, which is inconsistent with the finding in developed countries [44].

Finally, the based assumption of the staggered Difference-in-Difference method is the parallel trends between the HSR-affected universities in the treatment group and unaffected universities in the control group [27,28], suggesting that there is no significant difference in the value trend of the dependent variable between the treatment group and the control group. Following Wang et al [27] and Zhang et al [28], we regress model(1) with the dynamic dummies to formally test the parallel trend hypothesis. Specifically, we generate several years of dummy variables Forth_1, Forth_2, Current, Back_1, and Back_2. Forth_1 is a dummy variable that equals one if a university-year observation is from one year before the HSR opening and 0 otherwise. Forth_2 is a dummy variable that equals one if a university-year observation is from two years before the HSR opening and 0 otherwise. Current is a dummy variable that equals one if a university-year observation is from the opening year(year 0) and 0 otherwise. Back_1 is a dummy variable that equals one if a university-year observation is from one year after HSR opening and 0 otherwise. Back_2 is a dummy variable that equals one if a university-year observation is from two years after HSR opening and 0 otherwise. If the parallel trend assumption is satisfied, the coefficient of Forth_1 and Forth_2 are not all significant. As shown in column 5 of Table 4, we find that the hypothesis is satisfied.

### 4.5 Mechanism tests

In this subsection, we explore the possible mechanisms through which HSR affects university technology transfer. We examine two plausible channels, namely, the interaction channel and the demand channel. Testing these channels is challenging in our setting because not all channels are easily observable and measurable in the data. Hence, we expect to provide suggestive evidence that helps advance our understanding of the two channels.

Interaction about tacit knowledge between inventors in universities and enterprises is unobservable in data, so we cannot develop a direct measure to capture the channel in our setting. If it is interaction about tacit knowledge between inventors in universities and enterprises that drives our finding, we should expect to observe more cooperation between the two parties because cooperation means that supplier and consumer have more interaction [61]. Therefore, we use the cooperation level to reflect the interaction between the two parties. We use the research funding from enterprises divided by total research funding to capture the interaction between inventors in universities and enterprises(Interaction). In terms of the demand channel, we use the natural logarithm of the number of larger enterprises in a prefecture city where university i is located because more enterprise means more technology demand for the university(Demand).

Following Ye et al [62], we use the following mode to investigate the channel that HSR affects university technology transfer.


$$\begin{array}{cc} {Internation/Demand}_{i,t} & =\gamma_{0}+\gamma_{1}{HSR}_{i,t}+\gamma_{3}{Treat}_{i}+\gamma_{3}{Resp}_{i,t}\\ & +\gamma_{4}{Type}_{i,t}+\gamma_{5}{Expenditure}_{i,t}{+\gamma }_{6}{ADGP}_{i,t}\\ & +\gamma_{7}{Digital}_{i,t}+\eta_{j}+\mu_{t}+\varepsilon_{i,t} \end{array}$$ (2)

$$\begin{array}{cc} {Transfer}_{i,t} & =\alpha_{0}+\alpha_{1}{HSR}_{i,t}+\alpha_{2}{Treat}_{i}+\alpha_{3}{Resp}_{i,t}+\alpha_{4}{Type}_{i,t}\\ & +\alpha_{5}{Expenditure}_{i,t}+\alpha_{6}{AGDP}_{i,t}+\alpha_{7}{Digital}_{i,t}\\ & +\alpha_{8}{Interaction/Demand}_{i,t}+\eta_{j}+\mu_{t}+\varepsilon_{i,t} \end{array}$$ (3)

Where i denotes university and t denotes year. In model (2), the interest of the coefficient is γ_1_. In model (3), the interest of coefficients is α_1_ and α_8_. If HSR can affect university technology transfer through the interaction channel and the demand channel, the coefficient of γ_1_, α_1_, and α_8_ are significantly positive, and the value coefficient of α_8_ or the significance of α_8_ is lower than that in column 3 of Table 5.

**Table 5:** The table reports the result of robustness tests.

	(1)	(2)	(3)	(4)	(5)
	Transfer	Transfer	Trenafer1	Transfer	Transfer
HSR	0.087*	0.143**	0.363***	0.175***	
	(1.739)	(2.460)	(3.107)	(3.733)	
Treat	0.411***	0.257***	0.626***	0.206***	0.301***
	(4.583)	(3.873)	(4.935)	(4.418)	(4.451)
Resp	0.572***	0.473***	1.137***	0.405***	0.405***
	(34.395)	(23.171)	(37.039)	(33.182)	(33.145)
Type	0.182***	0.749***	1.908***	0.868***	0.863***
	(4.614)	(12.046)	(15.332)	(15.637)	(15.526)
Expenditure	-0.00100	0.0640	0.127	0.0660	0.0430
	(-0.003)	(0.198)	(0.185)	(0.244)	(0.159)
AGDP	0.0670	0.0330	-0.682	-0.193	-0.168
	(0.221)	(0.090)	(-0.976)	(-0.680)	(-0.591)
Digital	-0.240	-0.380*	-0.285	-0.241	-0.232
	(-1.174)	(-1.693)	(-0.700)	(-1.491)	(-1.422)
Law				0.458	
				(1.281)	
Forth_1					-0.0390
					(-0.489)
Forth_2					-0.144**
					(-2.103)
Back_1					0.0270
					(0.339)
Back_2					0.139**
					(2.065)
Constant	-2.980	-0.196	4.668	1.767	1.633
	(-1.293)	(-0.070)	(0.804)	(0.753)	(0.693)
Province fixed effect	Yes	Yes	Yes	Yes	Yes
Year fixed effect	Yes	Yes	Yes	Yes	Yes
N	6114	4709	6114	6114	6114
R^2^		0.304	0.373	0.352	0.353

In column 1, we change the regression method. In column 2, we exclude these universities without technology transfer in our sample period. In column 3, we use the natural logarithm of one plus transfer incomes to measure university technology transfer. In column 4, we consider the impact of the new Scientific and Technological Progress Law on university technology transfer. In the last column, we test the parallel trend assumption of the staggered Difference-in-Difference. The sample period is from 2007 to 2017. The t-statistics are reported in parentheses. The symbols *, **, and *** denote significant levels at the 10%, 5%, and 1%, respectively.

As shown in Table 6, we use mode (2) to examine the impact of HSR on the interaction between inventors in universities and enterprises in column 1. The coefficient of HSR is positive and significant at 5%, suggesting that HSR promotes the interaction between inventors in universities and enterprises. In column 2, we further use model (3) to test whether HSR affects university technology transfer by the interaction channel. We can find that the coefficient of HSR is significantly positive and the value of the coefficient is lower than that in column 3 of Table 5, and the coefficient of Interaction is positive and significant at the 1% statistical level. The results show that HSR can stimulate university technology transfer by promoting the interaction between inventors in universities and enterprises.

**Table 6:** The table reports the result of the mechanical test.

	(1)	(2)	(3)	(4)
	Interaction	Transfer	Demand	Transfer
HSR	0.016**	0.151***	0.347***	0.116**
	(2.161)	(3.283)	(17.428)	(2.384)
Treat	0.068***	0.106**	0.534***	0.114**
	(8.388)	(2.291)	(17.533)	(2.379)
Resp	0.036***	0.352***	0.064***	0.394***
	(16.752)	(29.109)	(12.019)	(32.015)
Type	-0.0100	0.883***	0.059***	0.858***
	(-1.379)	(16.582)	(3.861)	(15.450)
Expenditure	0.0460	-0.00100	0.383***	0.00
	(1.035)	(-0.005)	(3.290)	(0.002)
AGDP	-0.0180	-0.166	0.890***	-0.346
	(-0.387)	(-0.603)	(7.534)	(-1.218)
Digital	-0.0330	-0.192	0.403***	-0.310*
	(-1.321)	(-1.221)	(6.003)	(-1.917)
Interaction		1.474***		
		(17.696)		
Demand				0.172***
				(5.935)
Constant	0.0710	1.662	-6.837***	2.943
	(0.177)	(0.733)	(-7.016)	(1.262)
Province fixed effect	Yes	Yes	Yes	Yes
Year fixed effect	Yes	Yes	Yes	Yes
N	6114	6114	6114	6114
R^2^	0.179	0.389	0.800	0.355

In column 1 and column 2, we test whether HSR affects university technology transfer through the interaction channel. In columns 3 and column 4, we test whether HSR affects university technology transfer by the demand channel. The sample period is from 2007 to 2017. The t-statistics are reported in parentheses. The symbols *,**, and *** denote significant levels at the 10%,5%, and 1%, respectively.

In column 3 of Table 6, we use mode(2) to investigate the effect of HSR on the enterprises’ technology demand for universities. We can find that the coefficient of HSR is positive and significant at the 1% statistical level, indicating that HSR improves enterprises’ technology demand for universities. In column 4, we further use model (3) to test whether HSR affects university technology transfer by the demand channel. The coefficient of HSR is significantly positive, and the value of the coefficient is lower than that in column 3 of Table 5, and the coefficient of Demand is positive and significant at the 1% statistical level. In summary, the results suggest that HSR can stimulate university technology transfer by improving enterprises’ technology demand for universities.

### 4.6 Further analysis

There is significant heterogeneity in the development of institutions and the economy across China. In this subsection, we examine whether regional characteristics affect the relationship between HSR and university technology transfer.

#### 4.6.1 The effect of intellectual property protection

Although the law of intellectual property protection(IPR) is in accordance with international standards and is the same across China, the enforcement of IPR law has significant variations across regions [63]. For enterprises that purchase patents from universities, better intellectual property protection reduces the risk that innovation outputs are appropriated by other enterprises, which increases the benefit from new products. Thus, stronger intellectual property protection increases the incentive for enterprises to obtain patent licenses from universities, thus strengthening the effect of HSR on university technology transfer. We collect data on intellectual property infringement in the province from the China Nation Intellectual Property Administration. Then, we use the cumulative number of closed cases of intellectual property infringement divided by the cumulative number of filed cases of intellectual property infringement to measure intellectual property protection (IPR). Then, following Chang et al [64], we partition the sample according to the median value of IPR, namely, the high IPR group and low IPR group, and examine whether the relationship between HSR and university technology transfer has a difference in the two sub-samples.

As shown in Column 1 and Column 2 of Table 7, the coefficient of HSR is only positive significance in the high IPR group, suggesting that the association between HSR and university technology transfer is more prominent in regions with better intellectual property protection.

**Table 7:** Further analysis.

	High IPR	Low IPR	High Market	Low Market
	Transfer	Transfer	Transfer	Transfer
HSR	0.306***	0.0590	0.107	0.214***
	(4.373)	(0.906)	(1.445)	(3.320)
Treat	-0.0460	0.401***	0.298***	0.168***
	(-0.605)	(6.675)	(3.691)	(2.955)
Resp	0.414***	0.401***	0.459***	0.359***
	(22.861)	(24.145)	(24.812)	(22.153)
Type	0.759***	1.011***	0.987***	0.705***
	(9.995)	(12.380)	(13.376)	(8.414)
Expenditure	0.380	0.0650	-0.224	0.0710
	(0.889)	(0.167)	(-0.571)	(0.140)
AGDP	-0.610	0.00300	0.522	-0.723*
	(-1.519)	(0.006)	(1.088)	(-1.656)
Digital	-0.0400	-0.319	-0.407	-0.0310
	(-0.178)	(-1.300)	(-1.088)	(-0.141)
Constant	2.869	-0.0320	-3.332	4.698
	(0.836)	(-0.009)	(-0.832)	(1.248)
Province fixed effect	Yes	Yes	Yes	Yes
Year fixed effect	Yes	Yes	Yes	Yes
N	3066	3048	3056	3058
R^2^	0.344	0.366	0.366	0.318

This table shows the results of further analysis. In column 1 and column 2, we examine the impact of intellectual property protection on the relationship between HSR and university technology transfer. In column 3 and column 4, we examine whether the development of the technology trade market impacts the relationship between HSR and university technology transfer. The sample period is from 2007 to 2017. The t-statistics are reported in parentheses. The symbols *,**, and *** denote significant levels at the 10%,5%, and 1%, respectively.

#### 4.6.2 The effect of technology trade market

There is also considerable heterogeneity in the mature technology trade market in China. A more mature technology trade market, such as professional technology transaction agencies, suggests that universities have more channels to sell innovation outputs. This also means that HSR has less effect on university technology transfer. Therefore, we expect that the impact of HSR on university technology transfer will be more prominent in regions with underdevelopment of the technology trade market. We use the natural logarithm of the expenditure of technology transfer in a province to measure the maturity of the technology trade market(Market). Then, we partition the sample according to the median value of the Market, namely, the high market mature group and low market mature group, and examine whether the relationship between HSR and university technology transfer has a difference in the two sub-samples.

As shown in Column 3 and Column 4 of Table 7, the coefficient of HSR is only positive significance in the low market mature group, suggesting that the association between HSR and university technology transfer is more prominent in regions with underdevelopment of technology trade market.

## 5 Conclusion

University technology transfer is attracting more and more attention from policymakers and the academic community. Against the background of large-scale HSR construction in China, this paper examines the impact of HSR on university technology transfer for the first time. Using a total of 6114 university-year observations representing 732 universities from 2007 to 2017 and the staggered Difference-in-Difference method, we find that HSR has a positive effect on university technology transfer. The finding remains valid after changing the regression method, excluding universities without technology transfer in the sample period, replacing the measure of university technology transfer, controlling the impact of the new Scientific and Technological Progress Law, and testing the parallel trend assumption of the staggered Difference-in-Difference method. Mechanism tests find that HSR can stimulate university technology transfer by promoting the interaction between inventors in universities and enterprises and improving enterprises’ technology demand for universities. Further analysis suggests that better intellectual property protection strengthens the effect of high-speed rail on university technology transfer, and the relationship between high-speed rail and university technology transfer is more prominent in the regions with underdevelopment technology trading markets.

The evidence provided in this paper should be of interest not only to scholars but also to policymakers, given the fact that the consequence of university technology transfer is important for economic development. To stimulate university technology transfer, the government can expand investment in HSR, making more universities access to the HSR system. Besides, the government can consider providing more helps for enterprises with demand for university technology universities, such as tax incentives or subsidies. Finally, universities should offer more convenience for interaction between inventors in universities and enterprises, promoting the mobility of tacit knowledge between the two parties.

Notably, this paper provides indirect mechanisms to explain how HSR affects university technology transfer because some data fail to be obtained. Further research can use the survey data to provide more direct tests to identify how HSR affects university technology transfer. Besides, the finding of this paper may be appropriate for China. Other economies, including developed countries and developing countries, have different institutions and cultures. Thus, HSR may have a different effect on university technology transfer in these countries. Therefore, further analysis can be examined in other countries with HSR.

## Supporting information

S1 Data (DTA)
